# Highly accurate prediction of flammability limits of chemical compounds using novel integrated hybrid models

**DOI:** 10.1371/journal.pone.0224807

**Published:** 2019-11-14

**Authors:** Mohanad El-Harbawi, Brahim Belhaouari Samir, Lahssen El blidi, Ouahid Ben Ghanem

**Affiliations:** 1 Department of Chemical Engineering, King Saud University, Riyadh, Saudi Arabia; 2 Division of Information & Computing Technology, College of Science and Engineering, Hamad Bin Khalifa University, Doha, Qatar; 3 Department of process plant operations, Qatar Technical, Doha, Qatar; 4 Chemical Engineering Department, Universiti Teknologi PETRONAS, Bandar Seri Iskandar, Tronoh, Perak, Malaysia; Dartmouth College, UNITED STATES

## Abstract

Two novel and highly accurate hybrid models were developed for the prediction of the flammability limits (lower flammability limit (LFL) and upper flammability limit (UFL)) of pure compounds using a quantitative structure–property relationship approach. The two models were developed using a dataset obtained from the DIPPR Project 801 database, which comprises 1057 and 515 literature data for the LFL and UFL, respectively. Multiple linear regression (MLR), logarithmic, and polynomial models were used to develop the models according to an algorithm and code written using the MATLAB software. The results indicated that the proposed models were capable of predicting LFL and UFL values with accuracies that were among the best (i.e. most optimised) reported in the literature (LFL: *R*^2^ = 99.72%, with an average absolute relative deviation (AARD) of 0.8%; UFL: *R*^2^ = 99.64%, with an AARD of 1.41%). These hybrid models are unique in that they were developed using a modified mathematical technique combined three conventional methods. These models afford good practicability and can be used as cost-effective alternatives to experimental measurements of LFL and UFL values for a wide range of pure compounds.

## Introduction

Flammability can be broadly defined as the ease with which a material can be burned or ignited under specific conditions. The parameters-of-concern frequently used to characterise the flammability of chemical substances include the flash point, autoignition temperature, limiting oxygen concentration, lower flammability limit (LFL), and upper flammability limit (UFL) [1]. According to the American Society for Testing and Materials (ASTM), the LFL and UFL are defined as the lowest and highest concentrations (percentage) of the fuel (gas or vapor) in air capable of propagating a flame [2]. Flammability limits are commonly expressed using units of volume percent [3–7]. Most hydrocarbons are extremely volatile under relatively normal operating conditions [8–10]; thus, their flammability limits can be used to establish guidelines for the safe handling of these volatile substances. The flammability-limit values for pure compounds are typically found in material safety datasheets provided by the manufacturers. Extensive flammability data for pure gases and some gas mixtures can also be found in Bureau of Mines Bulletin publications [4–6, 11, 12] and elsewhere in the literature [13–15].

Many scientists performed experimental studies on flammability in the 1800s [3, 16] and 1900s [4–6, 11–13, 17]. Since then, several methods involving conventional experimental equipment, such as 20-L explosion apparatuses, have been introduced and utilised by numerous researchers to determine the flammability limits of gases and liquids [2, 18]. Shu and Wen [19] used this type of apparatus to investigate the flammability limits, maximum explosion overpressure, minimum oxygen concentration, and flammability zone of o-xylene. Chang et al. [20, 21] employed it to study the flammability of benzene and methanol with different vapor mixing ratios, as well as the flammability characteristics of 3-picoline/water mixtures. Liao et al. [22] conducted experiments to study the flammability limit of natural gas–air mixtures. Brooks and Crowl [23, 24] used the apparatus to study the flammability of vapours above aqueous solutions of ethanol and acetonitrile, as well as the flammability of methanol, ethanol, acetonitrile, and toluene mixtures. Wu et al. [25] employed a 20-L apparatus to investigate the flammability and explosion characteristics of methane with three different inert gases (CO_2_, N_2_, and Ar) at 1 atm and 30 or 100°C. Liaw et al. [26] used a 20-L spherical explosion vessel to study the flammability of a mixture containing acetone + steam, methanol + steam, methyl formate + steam, isopropyl alcohol + steam, isopropyl alcohol + nitrogen, and acetone + nitrogen.

Flammability limits can be determined using various established standard test methods: (i) ASTM methods (ASTM E681 and ASTM E918), (ii) the National Fire Protection Association method (NFPA 69), (iii) American Society of Heating, Refrigerating, and Air-Conditioning Engineers methods, and (iv) European methods (DIN 51649 and EN 1839). For details regarding these test methods, readers are referred to the work of Britton [27].

Even though these experimental standard tests are recommended for measuring the flammability limits of combustible gases, they are expensive and time-consuming. Additionally, because new chemicals are constantly being introduced in various industries, an easier and more cost-effective alternative to the experimental determination of flammability limits is needed. Scientists and engineers increasingly rely on desktop-based modelling methods for this purpose. One such method is the quantitative structure–property relationship (QSPR), which can quickly provide flammability-limit estimations with reasonable accuracy at a fraction of the cost and/or time of experimental testing.

QSPR studies have been widely applied for the prediction of the flammability limits of numerous substances. Many researchers have applied the QSPR for the estimation of LFL or UFL values. For example, Albahri [28] proposed models for estimating the flammability limits using the group contribution method. Gharagheizi developed different models for estimating the LFLs [29, 30] and UFLs [31, 32] of pure compounds using a QSPR method. Lazzús [33] employed an artificial neural network to predict the flammability limits of organic compounds according to their molecular structures. Rowley and Rowley [34] developed a method for predicting the LFLs of organic compounds via the group contributions approach and the heat of formation of the fuel. Bagheri et al. [35] suggested a model for the prediction of LFLs through a robust QSPR approach. Pan et al. [36–38] utilised the QSPR and developed models for estimating the flammability limits of organic compounds. Albahri [39] developed a neural network-based structural group contribution model for the prediction of LFLs. Frutiger [40] used the Marrero/Gani method to develop models for the prediction of LFLs, UFLs, flash points, and autoignition temperatures of organic chemicals. Chen et al. [41] proposed a QSAR model with four descriptors for predicting the LFLs of organic compounds. These models are compared in the “Results and discussion” section. Rowley [42] presented a comprehensive review of the use of QSPR models and other models for estimating LFL/UFL values.

In this study, we extracted 1057 LFL and 515 UFL data published by DIPPR Project 801 [14] to develop two new accurate models for predicting the LFLs and UFLs of pure compounds using the QSPR approach. These models were developed by combining three methods: multiple linear regression (MLR), logarithmic, and polynomial method. To the best of our knowledge, no QSPR model for the prediction of any property, including the LFL and UFL, based on a combination of these three methods has been reported in the literature.

## Materials and method

### Dataset collection and preparation

The dataset utilised in this study was obtained from the DIPPR Project 801 database [14]. The data were published by the American Institute of Chemical Engineers and can be considered as a reliable, comprehensive, and accessible source for the hazard and safety properties of pure compounds. The dataset covers a myriad of organic compounds with multiple functional groups, namely; hydrocarbons, halogenated hydrocarbon compounds, ethers, ketones, alcohols, aldehydes, amides, esters, amines, acids, nitriles, nitro compounds, and heterocyclic compounds. The first step in preparing the dataset was to design a ‘molecular structure table’ based on the molecular fragments (groups), for describing the molecular structure of the pure compounds. In this study, 1057 LFL values were selected from DIPPR Project 801 to develop the LFL model. The same dataset was previously used by Gharagheizi [29]. UFL values for 515 pure compounds were also selected from the DIPPR Project 801 database and used as the main UFL dataset (S1 and S2 Tables of the Electronic Supplementary Material). The molecular descriptors of all these pure compounds were determined using the software package *Dragon* [43]. This software is generally used for molecular descriptor calculations; details regarding its usage can be obtained from its website (http://www.talete.mi.it/) or from the Handbook of Molecular Descriptors [44]. The molecular descriptors (S1 and S2 Tables) were subsequently used as datasets for MATLAB processing to predict the best-fit models that afforded the most accurate predicted results (i.e. closest agreement between the experimental and predicted values). A brief description of the molecular descriptors used in this study is presented in S1 and S2 Tables).

### Model development

The QSPR process quantitatively correlates the structural properties of molecules (the descriptors) with their functional properties (in this case, the LFL and UFL values) for a set of similar compounds. The process uses linear statistical methods, such as MLR, polynomial regression, and partial least-squares, or nonlinear methods, such as support vector machines (SVMs), artificial neural networks (ANN), etc., to generate mathematical models that relate the experimentally measured properties of the compounds with a set of chemical descriptors.

In this study, we integrated MLR, logarithmic, and polynomial models to combine the inherent strengths of each model and enhance the predictive accuracy of the resultant model. In the case of linear regression, the dependent (prediction) variable was represented as *Y*, while the independent variables (descriptors) were represented as *X*_1_, *X*_2_, …, *X*_*p*_, where *p* represents the *p*^th^ predictor variable. The relationship between the response variable *Y* and the descriptors *X*_1_, *X*_2_, …, *X*_*p*_ can be expressed as a linear regression model (Eq (1)) [45]:

$$Y=f(X_{1},X_{2},\ldots \ldots ..X_{p})+\varepsilon ,$$ (1)

where *ε* represents the normal random error (residual) reflecting the difference between the observed and the predicted values. Eq (1) can be expressed in a linear form as

$$Y=\alpha_{0}+\alpha_{1}X_{1}+\alpha_{2}X_{2}+\ldots .+\alpha_{p}X_{p}+\varepsilon ,$$ (2)

where *a*_o_, *a*_1_, …, *a*_p_ are the regression coefficients for the MLR model. Eq (2) is in a linear form and can be expressed in a nonlinear form (i.e. logarithmic form):

$$Y=\beta_{1}\ln X_{1}+\beta_{2}\ln X_{2}+\ldots .+\beta_{p}\ln X_{p}+\varepsilon ,$$ (3)

where *β*_1_, *β*_2_, …, *β*_p_ are the regression coefficients for the logarithmic model.

The MLR (Eq (2)) and logarithmic model (Eq (3)) were then integrated with a polynomial model (Eq (4)) [46].

$$Y=\gamma_{0}+\gamma_{1}\omega^{1}+\gamma_{2}\omega^{2}+\ldots .+\gamma_{m}\omega^{m}+\varepsilon$$ (4)

This interaction yielded the final hybrid model) Eq (5)):

$$Y=\alpha_{0}+\sum_{i=1}^{n}\alpha_{i}X_{i}+\sum_{i=1}^{m}\gamma_{i}\omega^{1}+\sum_{i=n+1}^{k}\delta_{i}\omega X_{i}+\sum_{i=1}^{n}\beta_{i}\;\ln X_{i}+\sum_{j=1}^{n}\sum_{i=1}^{n}\lambda_{j,i}X_{j}\;\ln X_{i},$$ (5)

where

*n* represents the number of parameters for the MLR model,

*m* represents the number of parameters for the polynomial model,

*k* represents the number of interactions between the MLR model and the polynomial model,

$\sum_{i=n+1}^{k}\delta_{i}\omega X_{i}$ represents the interactions between the MLR and polynomial models, and

$\sum_{j=1}^{n}\sum_{i,=1}^{n}\lambda_{j,i}X_{j}lnX_{i}$ represents the interactions between the MLR and logarithmic models.

The parameters for the MLR model are *ω* = ∑*X*_*i*_, *α*_*i*_ = *β*_*i*_ for *i* ≤ *n*, *γ*_*i*_ = *β*_*i*+*n*_ for *i* ≤ *m*, *δ*_*i*_ = *β*_*i*+*n*+*m*_ for *i* ≤ *k*, and 2*n*+*m*+*k*+*n*^2^ = *p*, *α*_0_,*α*_1_,……*α*_*n*_. *γ*_1_……*γ*_*m*_. *β*_1_…… *β*_*n*_ are the parameters for the logarithmic model. λ_*0*,*0*_,λ_i,i_,……λ_n,n_ and *δ*_1_……*δ*_*k*_ are the parameters for the interactions between the descriptors.

The interaction between the polynomial and logarithmic models was found to have a negligible effect on the results of the proposed model.

To estimate the overall parameters of the proposed model, we used the least-squares error method. The corresponding prediction equation is

$$\overset{⌢}{Y}=\alpha_{0}+\sum_{i=1}^{n}{\overset{⌢}{\alpha }}_{i}X_{i}+\sum_{i=1}^{m}{\overset{⌢}{\gamma }}_{i}\omega^{i}+\sum_{i=n+1}^{k}{\overset{⌢}{\delta }}_{i}\omega X_{i}+\sum_{i=1}^{n}{\overset{⌢}{\beta }}_{i}lnX_{i}+\sum_{j=1}^{n}\sum_{i=1}^{n}{\overset{⌢}{\lambda }}_{j,i}X_{j}lnX_{i}$$ (6)

The algebraic matrix for the proposed models is given as follows:

$$\overset{⌢}{Y}=\Psi •\overset{⌢}{\Theta },$$ (7)

where

$$\overset{⌢}{\Theta }=\left({\overset{⌢}{\alpha }}_{0},{\overset{⌢}{\alpha }}_{1}\ldots \ldots {\overset{⌢}{\alpha }}_{n},{\overset{⌢}{\gamma }}_{1},\ldots \ldots {\overset{⌢}{\gamma }}_{m},{\overset{⌢}{\delta }}_{1},\ldots \ldots {\overset{⌢}{\delta }}_{k},{\overset{⌢}{\gamma }}_{1},\ldots \ldots {\overset{⌢}{\gamma }}_{m},{\overset{⌢}{\beta }}_{1},\ldots \ldots {\overset{⌢}{\beta }}_{n},{\overset{⌢}{\lambda }}_{1,1},\ldots \ldots {\overset{⌢}{\lambda }}_{n,n}\right)={\left(\Psi '\Psi \right)}^{-1}\Psi 'Y$$

and

$$\Psi =\left[\begin{array}{lllll} X_{1,1}\ldots \ldots X_{n,1} & \omega_{1} & \omega X_{1,1}\ldots \ldots ..\omega X_{n,1} & \ln X_{1,1}\ldots \ldots ..\ln X_{n,1} & X_{1,1}\ln X_{1,n}\ldots \ldots \ldots \ldots X_{n,1}\ln X_{n,1}\\ .\;. & . & .\;. & .\;. & .\;.\\ .\;. & . & .\;. & .\;. & .\;.\\ .\;. & . & .\;. & .\;. & .\;.\\ X_{1,N}\ldots \ldots X_{n,N} & \omega_{N} & \omega X_{1,N}\ldots \ldots ..\omega X_{n,N} & \ln X_{1,N}\ldots \ldots ..\ln X_{n,N} & X_{1,N}\ln X_{1,N}\ldots \ldots \ldots X_{n,N}\ln X_{n,N} \end{array}\right]$$

Here, *N* represents the number of LFL/UFL experimental values for the pure compounds.

**Fig 1:**
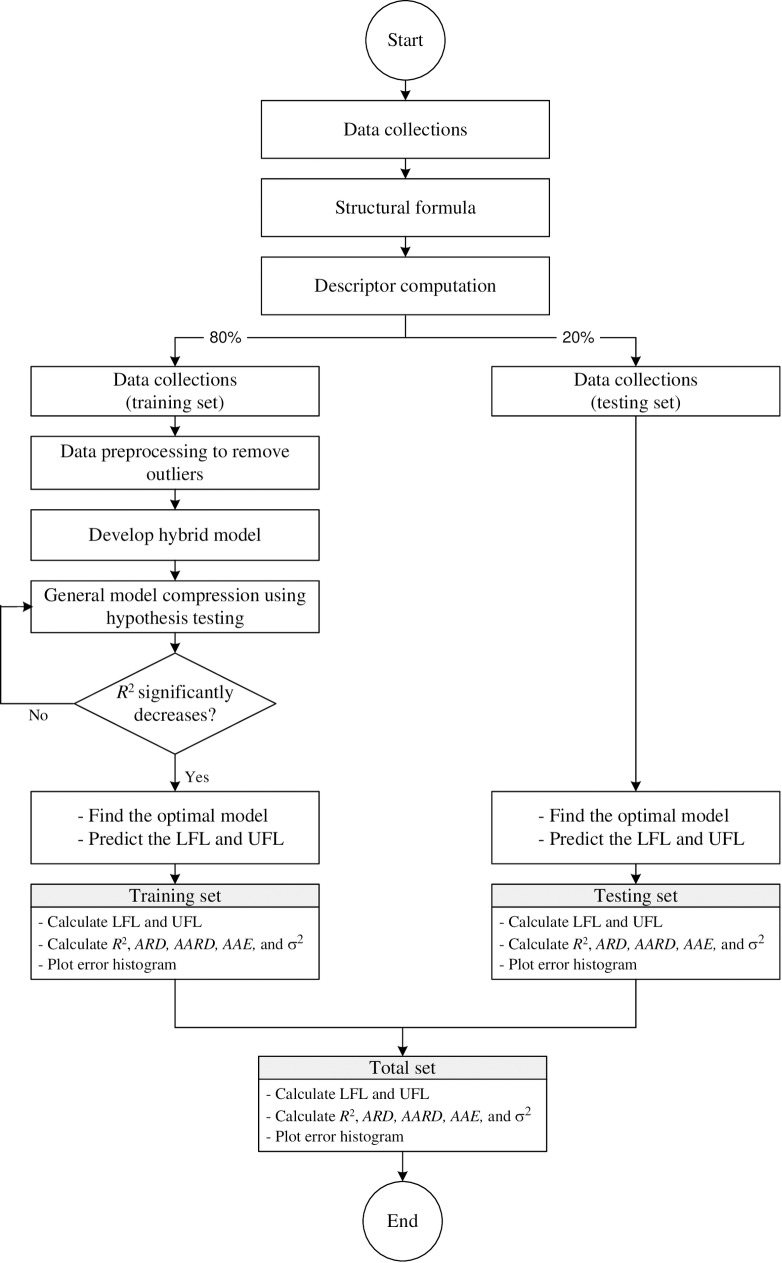
Algorithm for the prediction of the LFL and UFL using MATLAB.

The MATLAB software (version 7.8.0.347) was employed to build the code and predict the LFL and UFL values using the algorithm shown in Fig 1.

The average relative deviation (ARD, Eq (8)), average Absolute relative deviation (AARD, Eq (9)), average absolute error (AAE, Eq (10)), and standard deviation (square root of the variance, ${\hat{\sigma }}^{2}$, Eq (11)) were used to confirm the accuracy of the developed model.

$$ARD=\frac{100}{N}{\sum_{i=1}^{N}\left(\frac{FL_{Cal}-FL_{Exp}}{FL_{Exp}}\right)}_{i}$$ (8)

$$AARD=\frac{100}{N}\sum_{i=1}^{N}{\left|\frac{FL_{Cal}-FL_{Exp}}{FL_{Exp}}\right|}_{i}$$ (9)

$$AAE=\frac{100}{N}\sum_{i=1}^{N}{\left|FL_{Cal}-FL_{Exp}\right|}_{i}$$ (10)

$${\hat{\sigma }}^{2}=\frac{\sum_{i=1}^{N}{\left(FL_{Cal}-F{\bar{L}}_{Cal}\right)}_{i}^{2}}{N‑1}$$ (11)

Here,

*N* represents the number of substances,

*FL*_*Cal*_ represents the calculated flammability value (LFL or UFL),

*FL*_*Exp*_ represents the experimental flammability value (LFL or UFL), and

$F{\bar{L}}_{Cal}$ represents the mean *FL* value.

To determine the significant coefficients that define the relationship between the flammability limits of each compound and their molecular structures, the MLR, logarithmic, and polynomial models were combined via the group contribution method, as indicated by Eq (5). MATLAB was employed to perform the calculations. The code was written using an 80%/20% training/testing split. The purpose of the training process was to calibrate the model and to optimise the optimal coefficients according to the least-squares method. This method yields the best-fitting curve between the predicted results and the DIPPR 801 LFL/UFL values. The validation process was used for predicting the values not included in the training set. To reduce the number of coefficients in the final models without losing accuracy, *R*^2^ hypothesis testing was performed for each coefficient to evaluate its significance in the developed model. Coefficients with insignificant values were eliminated to simplify the models. Only the most significant coefficients obtained from the hypothesis testing were selected and used in Eq (5) to build the final models and then to predict the results.

## Results and discussion

The training set was initially subjected to the least-squares method for developing the different models. The MATLAB program utilised the DIPPR data (80% of the entire dataset) to train the code and then to compute the coefficients for the developed models (*α*_*i*_,*γ*_*i*_,*δ*_*i*_,*λ*_*i*,*j*_) using Eq (5). The MATLAB code then analysed the remaining data (20% of the entire dataset) using the coefficients obtained from the training dataset to evaluate how well the models had been trained and how accurately the models could predict the results. The testing set was not used during the training process and was only used to compare the predicted results. For the development of the LFL model, 846 components were utilised for the training set, and 211 were used for the testing set. The LFL model was constructed according to the 105 molecular descriptors, as described in S1 Table. For the UFL model, 412 components were utilised for the training set, and 103 were used for the testing set. Furthermore, 82 molecular descriptors were used to build the UFL model (S2 Table). For the proposed method, the interactions among the three models generated a large number of coefficients, which enhanced the accuracy of the models. For instance, the number of coefficients for the LFL model was 6421, and the model had an *R*^2^ of 99.72%. For the UFL model, the number of coefficients was 12481, and the model had an *R*^2^ of 99.64%. It is highly recommended to use the proposed models with the aforementioned numbers of coefficients. This is because if the number of coefficients is reduced (e.g. from 6421 to 357 for LFL and from 12481 to 175 for UFL), the accuracy of the LFL and UFL models decreases (to *R*^2^ = 96% and *R*^2^ = 78%, respectively). Table 1 presents the comparison results for the accuracies of the three models interacting together (proposed models) and the three models individually.

**Table 1:** Comparison of the accuracy between the developed model and other models.

Model	*R*^2^ (%)	${\hat{\sigma }}^{2}$
**LFL**		
**MLR**	76.06	0.06
**Polynomial**	12.64	0.21
**Logarithmic**	52.36	0.11
**MLR + logarithmic + polynomial (proposed model)**	99.72	6.6 × 10^−4^
**UFL**		
**MLR**	84.73	1.06
**Polynomial**	74.30	1.95
**Logarithmic**	76.55	1.64
**MLR + logarithmic + polynomial (proposed model)**	99.64	0.041

### LFL prediction accuracy and validation

Table 2 presents the statistical parameters of the training, testing, and total datasets. The developed model was capable of predicting the LFL with a high accuracy (*R*^2^ = 99.69% for the training set, *R*^2^ = 99.83% for the testing set, and *R*^2^ = 99.72% for the whole dataset). Additionally, the *R*^2^, ARD, AARD, AAE, and ${\hat{\sigma }}^{2}$ values of the training and testing sets were very similar. This indicates that the predicting abilities of the proposed model were stable. To validate the proposed model, we tested the MLR, polynomial, and logarithmic models separately. The output accuracies (*R*^2^) of these methods were 76.06%, 12.64%, and 52.36%, respectively (Table 1). The proposed model (MLR + logarithm transformation) exhibited far more accurate prediction (*R*^2^ = 99.72%) than the individual models, indicating that the proposed concept of using a hybrid model based on the interaction between these three models enhanced the accuracy of the results. This is because the combination of the three models took more predictor variables (*X*_1_, *X*_2_, …, *X*_n_) into consideration during the processing of the data by the MATLAB code and optimised the coefficients (*α*_*i*_,*γ*_*i*_,*δ*_*i*_,*λ*_*i*,*j*_) for use in the best model. The most significant coefficients were optimised using MATLAB and applied to Eq (5) to obtain the best model for predicting the LFL.

**Table 2:** Statistical parameters of the developed models.

Statistical parameters	Training set	Testing set	Whole dataset
**LFL**			
**No. of compounds**	846	211	1057
***R***^***2***^ **(%)**	99.69	99.83	99.72
***ARD* (%)**	0.09	0.08	0.10
***AARD* (%)**	1.67	0.8	0.80
***AAE* (%)**	1.30	0.65	1.20
${\hat{\sigma }}^{2}$	6.7 × 10^−4^	7.8 × 10^−4^	6.6 × 10^−4^
**UFL**			
**No. of compounds**	412	103	515
***R***^***2***^ **(%)**	99.34	99.33	99.64
***ARD* (%)**	0.088	0.084	0.086
***AARD* (%)**	1.76	1.78	1.41
***AAE* (%)**	11.68	11.75	9.87
${\hat{\sigma }}^{2}$	0.033	0.033	0.041

**Fig 2:**
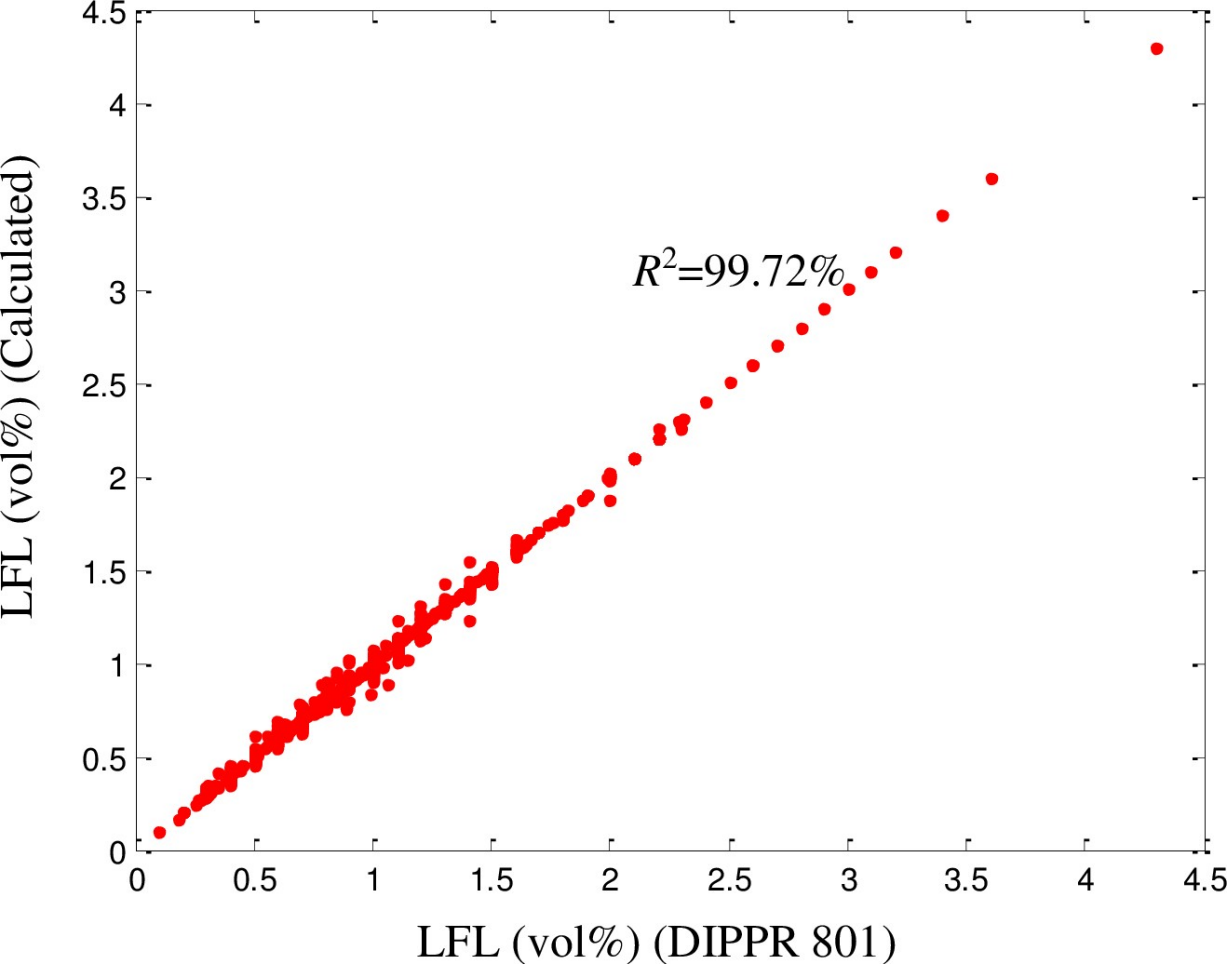
Comparison between predicted and DIPPR 801 LFL values.

**Fig 3:**
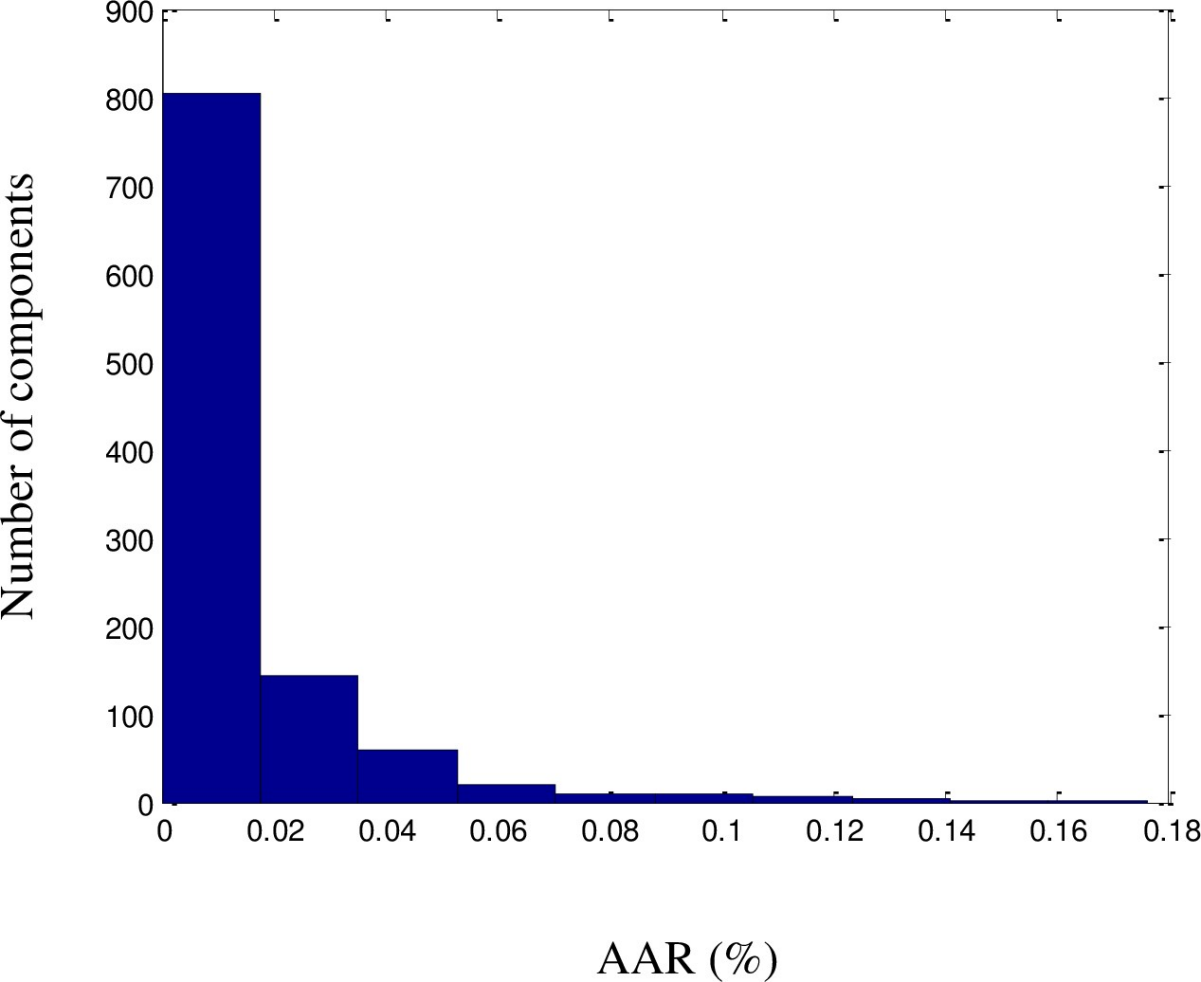
AAR histogram for the LFL model.

**Fig 4:**
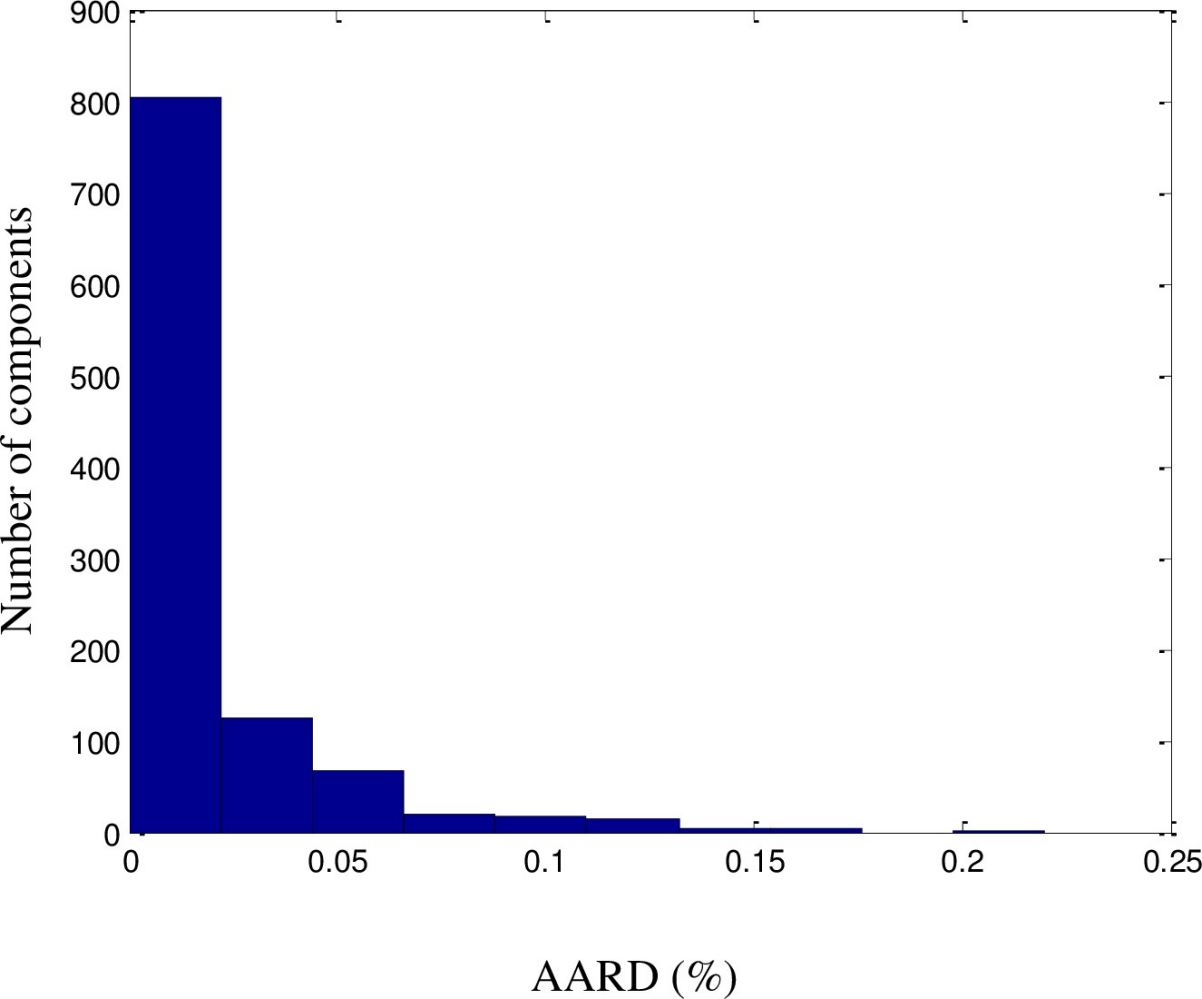
AARD histogram for the LFL model.

**Fig 5:**
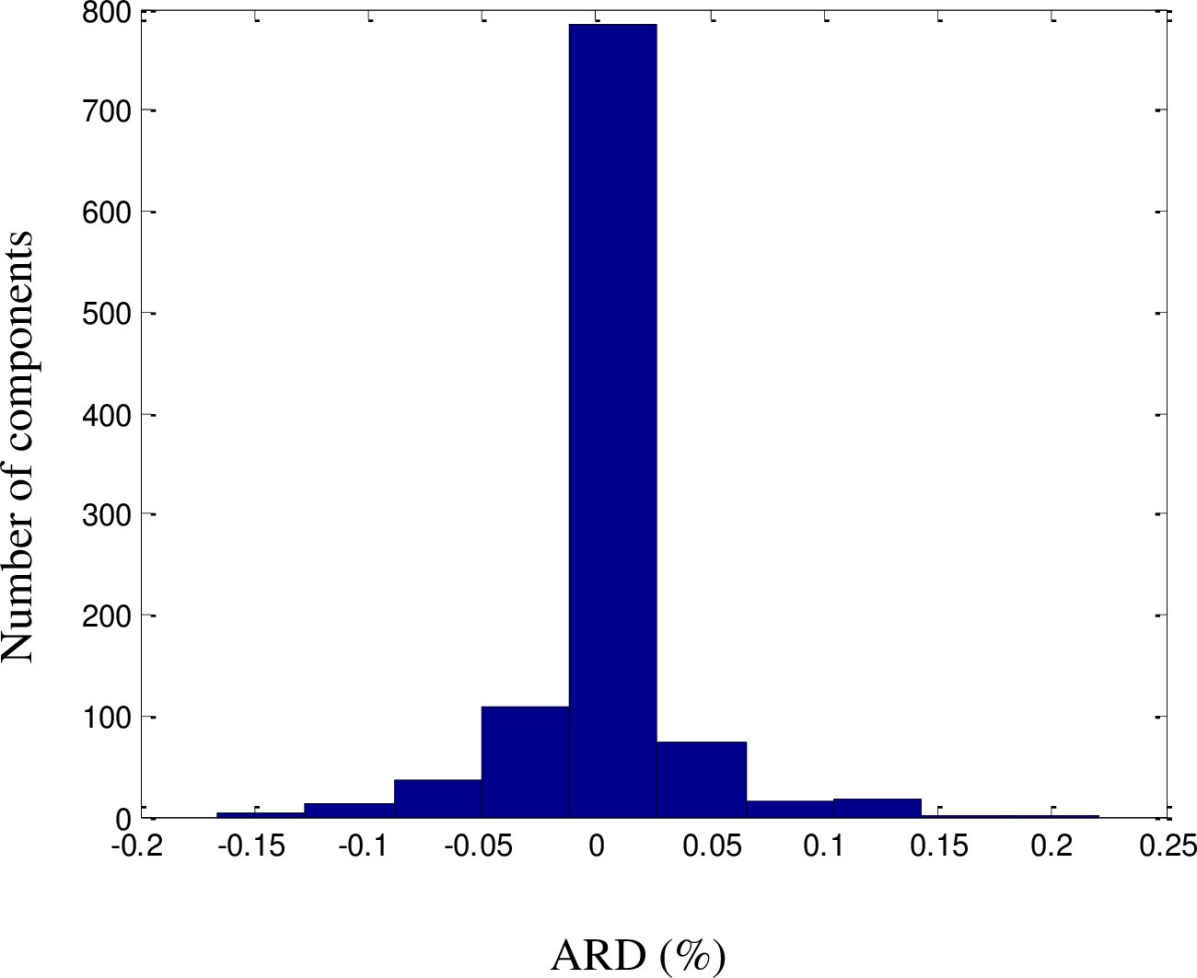
ARD histogram for the LFL model.

All QSPR models require further validation before they can be considered reliable. The proposed model was validated using a dataset consisting of a random selection of 20% of the components in the dataset. The predicted results were validated against the experimental values of the dataset and were found to be consistent, with no significant deviations. An excellent fit was achieved (*R*^2^ = 99.72%), as illustrated in Fig 2. The ARD, AARD, AAE, and standard deviation were 0.1%, 0.8%, 1.2%, and 6.6 × 10^−4^, respectively. As shown in Figs 3–5 among the 1057 components, there were approximately 800 components with ‘zero’ error between the predicted LFL values and DIPPR 801 values. The results predicted using the model were also compared with results obtained using models developed by other authors, as shown in Table 3.

**Table 3:** Comparison of several QSAR models and their accuracies.

Method	No. of Compounds	*R*^2^ (%)	AAE (%)	ARD (%)	AARD (%)	References
**LFL**						
**Artificial neural network**	454	93.00	4.1	-	-	Albahri [28]
**Genetic algorithm**	1056	97.90	5.0	-	-	Pan et al. [37]
**Genetic algorithm**	354	96.70	4.3	-	-	Pan et al. [38]
**Genetic algorithm multivariate linear regression**	1057	98.60	4.62	-	-	Gharagheizi [30]
**Artificial neural network**	509	91.70	10.76			Rowley and Rowley [34]
**Artificial neural network**	418	98.65	-	-	8.6	Lazzús [33]
**MLR**	458	91.4	0.3442	-	-	Chen et al. [41]
**MLR**	1615	90.61	0.186	-	-	Bagheri et al. [35]
**Neuro-fuzzy inference system**	1615	92.90	0.153	-	-	Bagheri et al. [35]
**Artificial neural network**	543	99.98	0.02	-	-	Albahri [39]
**Proposed model**	1057	99.72	1.2	0.1	0.80	This work
**UFL**						
**Artificial neural network**	464	92.2	11.8	-	-	Albahri [28]
**Genetic algorithm + MLR**	579	75.8	1.75	-	-	Pan et al. [36]
**Genetic algorithm + MLR**	354	96.7	4.3	-	-	Pan et al. [38]
**Genetic algorithm + multivariate linear regression**	865	92	9.7			Gharagheizi [31]
**Genetic algorithm + multivariate linear regression**	1294	95	-	3.56	25.76	Gharagheizi [32]
**Artificial neural network**	418	98.18	-	-	7.1	Lazzús [33]
**Proposed model**	515	99.64	9.87	0.086	1.41	This work

Albahri [39] developed a model for predicting the LFL with a higher accuracy than our model (*R*^2^ = 99.98%). However, the number of compounds used in his study (543) was smaller than that utilised in the present study (1057). To test the efficiency of our novel MATLAB code, we utilised the dataset provided by Pan et al. [37] and developed an accurate LFL model (Eq (12)). The results of Pan et al. [37] were compared with our results, as shown in S3 Table. Pan et al. [37] developed four different models. The support vector machine (SVM) model had the highest accuracy (*R*^2^ = 99.97%), which was equal to the accuracy of our model (Eq (12)). However, none of the four models of Pan et al. [37] were presented as a mathematical equation.

$$\begin{array}{l} LFL=17.1352\left(SIC0\right)‐1.8536\left(AAC+PW5\right)+1.044\left(AAC\right)\left(AAC+PW5\right)‐\\ \;11.266\left(SIC0\right)\left(AAC+PW5\right)‐76.4768{\left(PW5\right)}^{2}\left(AAC+PW5\right)+13.4096{\left(\mathrm{SIC}0\right)}^{2}\\ \;\left(\mathrm{AAC}+\mathrm{PW}5\right)+28.0829{\left(\mathrm{SIC}0\right)}^{2}\left(SIC0+GATS1v\right)+0.032{\left(SIC0\right)}^{3}\left(SIC0+GATS1v\right)+\\ \;518.4484\left(AAC+PW5\right){\left(PW5\right)}^{3}-9.1264\left(AAC+PW5\right){\left(SIC0\right)}^{3}-165.743\\ \;\left(PW5+SIC0\right){\left(PW5\right)}^{2} \end{array}$$ (12)

Here,

SIC0 represents information indices (structural information content, neighbourhood symmetry of 0-order),

AAC represents topological descriptors (mean information index on atomic composition),

PW5 represents topological descriptors (path/walk 5 Randic shape index), and

GATS1v represents two-dimensional (2D) autocorrelations (Geary autocorrelation—lag 1/weighted by atomic van der Waals volumes).

### UFL prediction accuracy and validation

**Fig 6:**
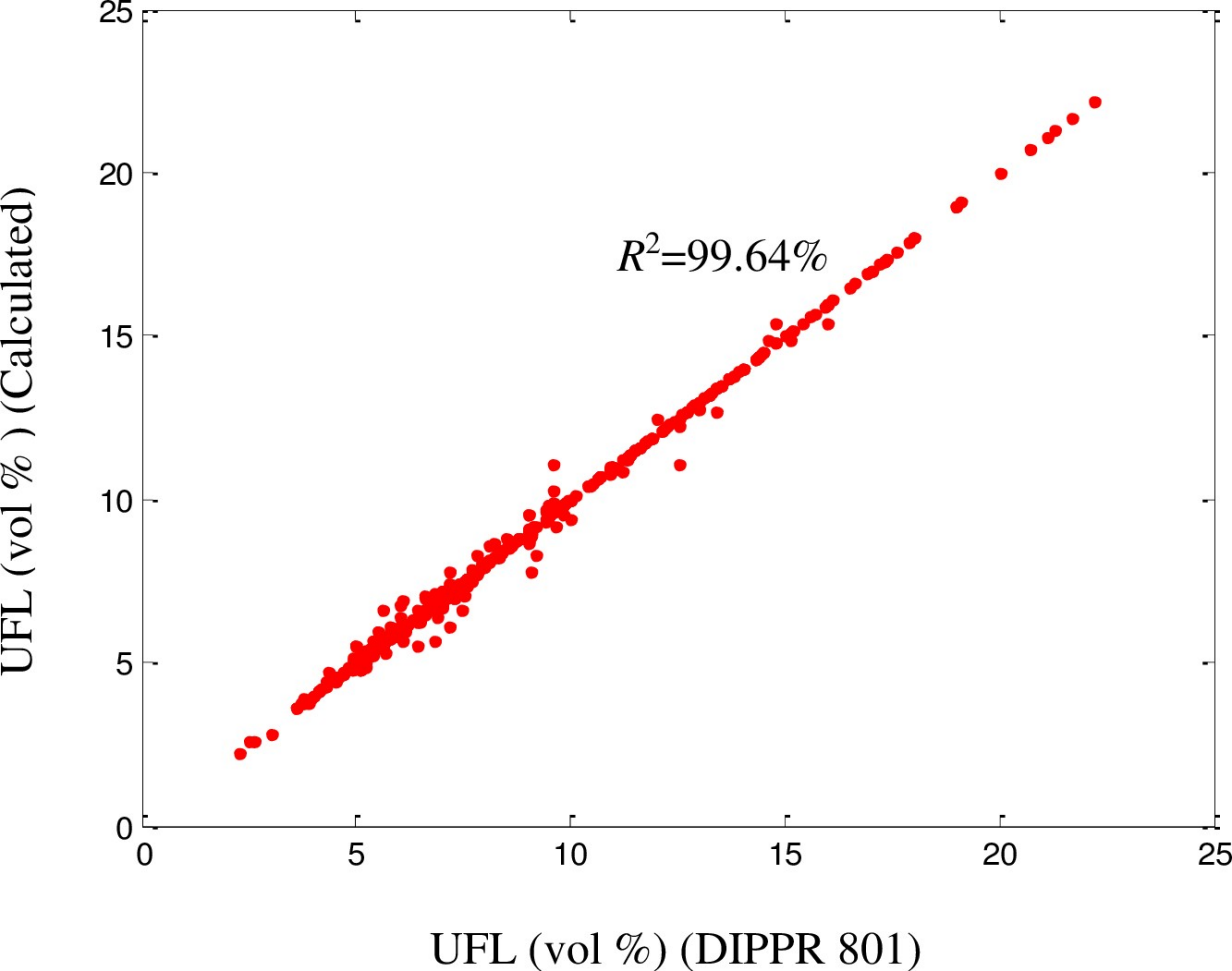
Comparison between predicted and DIPPR 801 LFL values.

**Fig 7:**
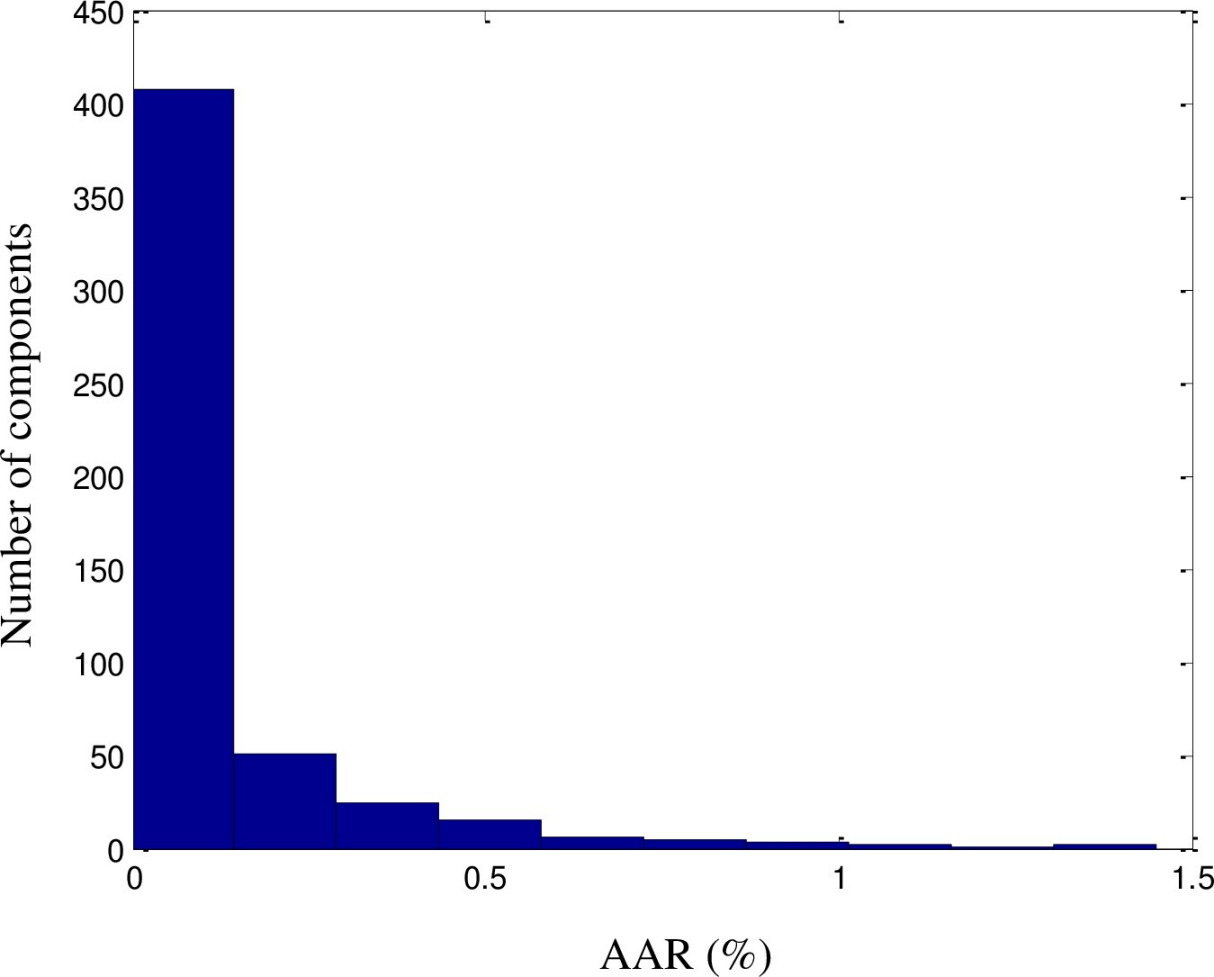
AAR histogram for the UFL model.

**Fig 8:**
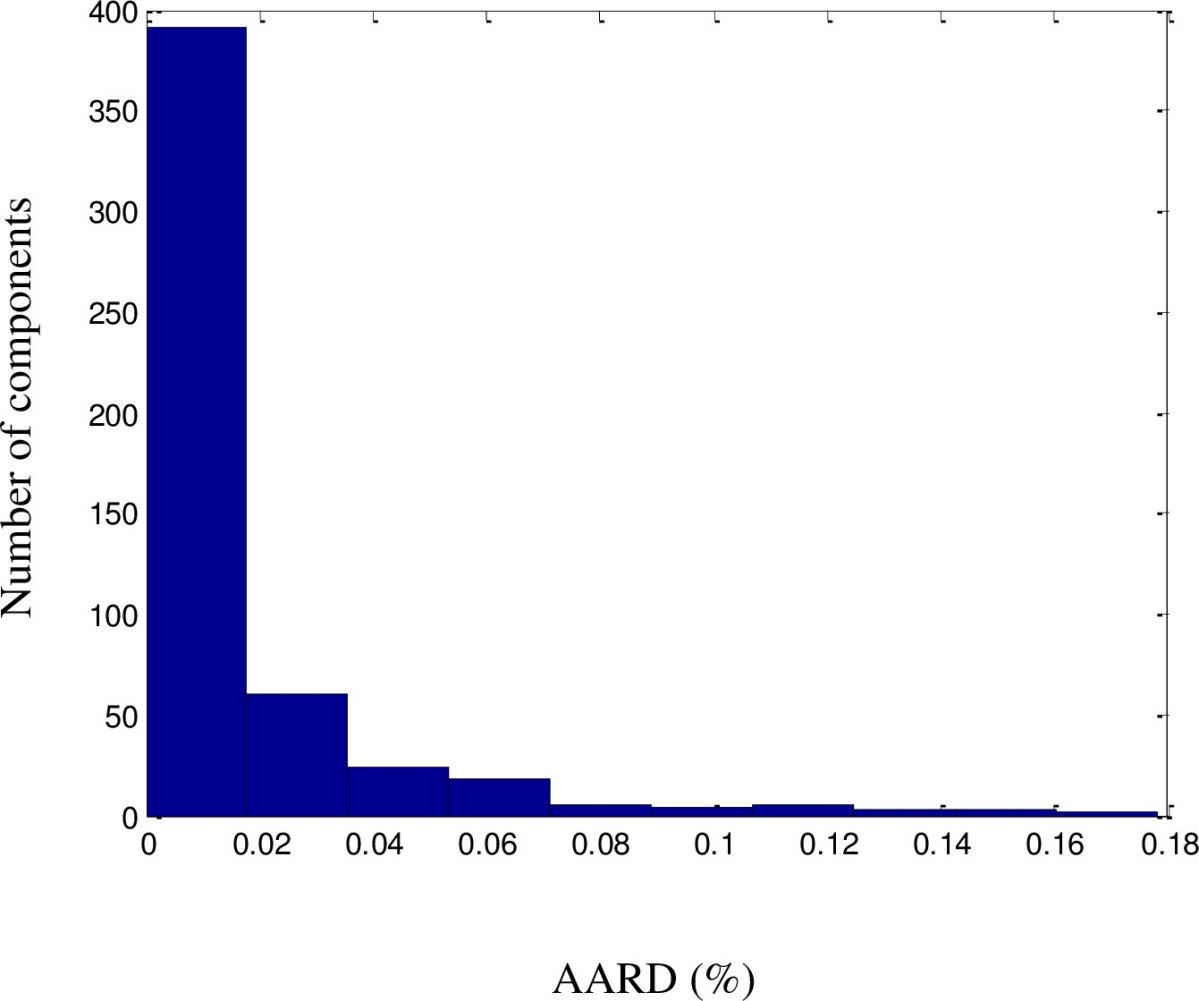
AARD histogram for the UFL model.

**Fig 9:**
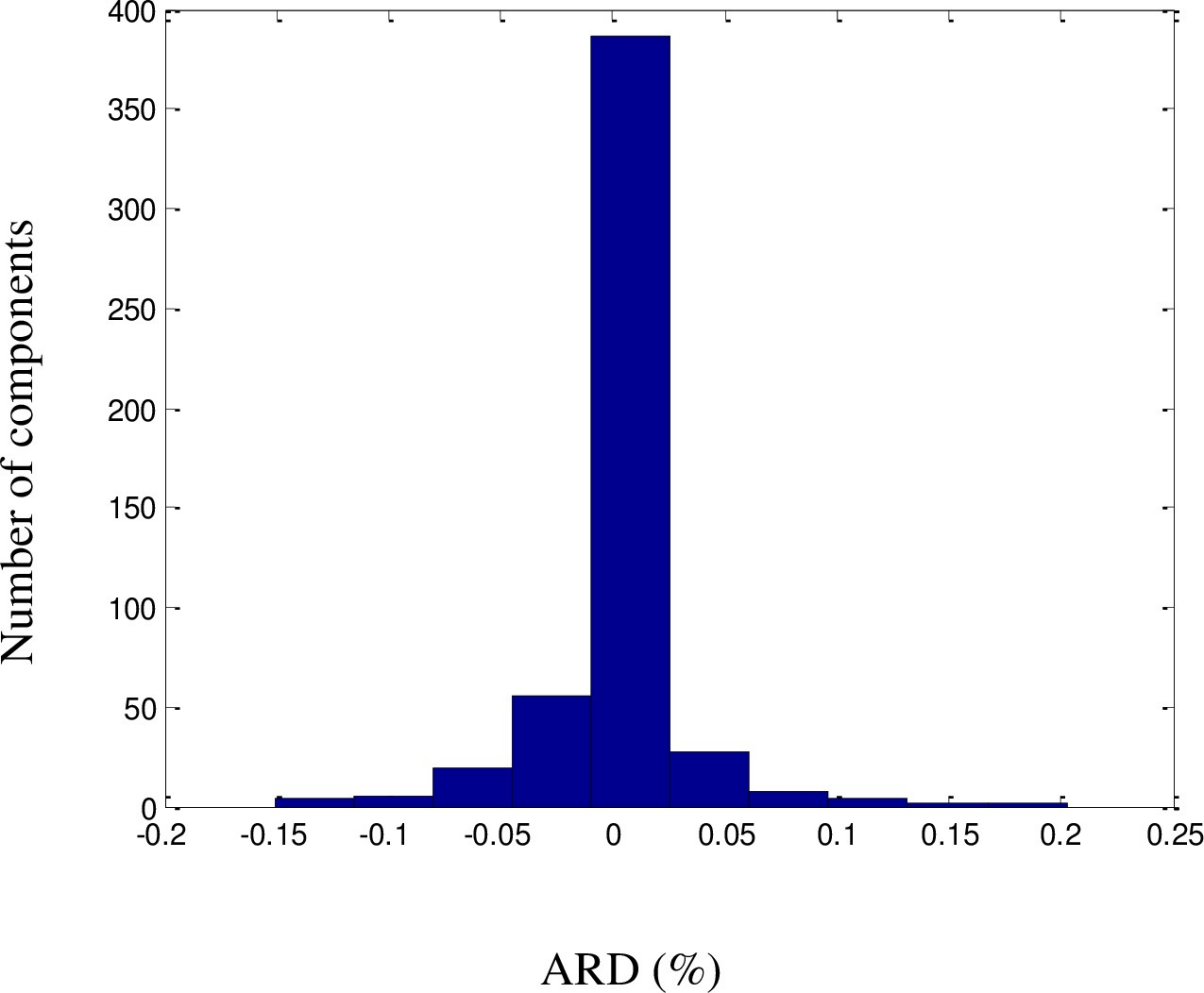
ARD histogram for the UFL model.

It can be clearly concluded from Table 2 that the developed model was able to predict the UFL values with a high accuracy (*R*^2^ = 99.34% for the training set, *R*^2^ = 99.33% for the testing set, and *R*^2^ = 99.64 for the whole dataset). The UFL values obtained using the proposed model were compared with the experimental values from DIPPR 801. A good fit was achieved (*R*^2^ = 99.64%), as illustrated in Fig 6. The ARD, AARD, AAE, and standard deviation were 0.086%, 1.41%, 9.87%, and 0.041, respectively. As shown in Figs 7–9,among the 515 components, approximately 470 exhibited ‘zero’ error between the predicted UFL values and the DIPPR 801 values. The results predicted by the model were also compared with results obtained using models developed by other authors, as shown in Table 3.

Gharagheizi [31] was able to predict a simple UFL equation with six coefficients using a dataset containing only five descriptors. The accuracy of his model was high (*R*^2^ = 0.9202). Additionally, Pan et al. [36] developed two simple UFL models with six and four coefficients based on the MLR method and a dataset containing only four descriptors. The accuracy of the two models were not high (*R*^2^ = 0.57 and 0.758 respectively). To test the accuracy of our developed algorithm and MATLAB code, we utilised Gharagheizi’s dataset [31] and developed a UFL equation (Eq (13)). The model predicted the UFL with an *R*^2^ of 92.72%. This indicates that the accuracy of the proposed model is slightly higher than that of Gharagheizi’s model [31]. Details are presented in S4 Table.

$$\begin{array}{l} UFL=14.011-0.765\;MLOGP-33.853\left(Jhetv+PW5\right)+0.834\left(SIC0+MATS4m\right)+\\ \;32.167\left(Jhetv-PW5\right)-281.86{\left(PW5\right)}^{2}+35.904{\left(SIC0\right)}^{2}+2622.185{\left(PW5\right)}^{3}-\\ \;23.301{\left(SIC0\right)}^{3}+11.134\left(Jhet+PW5\right)PW5 \end{array}$$ (13)

Here,

Jhetv represents topological descriptors (balaban-type index from van der Waals weighted distance matrix),

MATS4m represents 2D autocorrelations (Moran autocorrelation-lag 4 weighted by atomic masses), and

MLOGP represents molecular properties (Moriguchi octanol–water partition coefficient (log *P*)).

## Conclusion

A new method was proposed for the development of flammability-limit (LFL and UFL) models based on a QSAR approach. The development of these models was based on code written using the MATLAB software (version 7.8.0.347) and a combination of MLR, logarithmic, and polynomial models. To develop the LFL and UFL models, 1057 and 515 pure compounds were used, respectively, spanning many families of compounds. Therefore, the developed models have a wide range of applicability. The developed models predicted the LFL and UFL with high accuracy (*R*^2^ = 99.72% and *R*^2^ = 99.64%, respectively) and are more accurate than previously reported models.

## Supporting information

S1 Table Functional groups and detailed results for the LFL. (XLS)

S2 Table Functional groups and detailed results for the UFL. (XLSX)

S3 Table Results comparison for the Pan et al. [37] dataset. (XLS)

S4 Table Results comparison for the Gharagheizi [36] dataset. (XLS)
