# Correction: Highly accurate prediction of flammability limits of chemical compounds using novel integrated hybrid models

**DOI:** 10.1371/journal.pone.0229780

**Published:** 2020-02-21

**Authors:** Mohanad El-Harbawi, Brahim Belhaouari Samir, Lahssen El blidi, Ouahid Ben Ghanem

The first author, Mohanad El-Harbawi, is incorrectly noted as the only corresponding author. The second corresponding author is Brahim Belhaouari Samir. Dr. Samir’s email address is: sbelhaouari@hbku.edu.qa

Additionally, the following information is missing from the Funding statement: Support for this study was provided by the Qatar National Library to BBS.
