# Correction: Highly accurate prediction of flammability limits of chemical compounds using novel integrated hybrid models

**DOI:** 10.1371/journal.pone.0303936

**Published:** 2024-05-15

**Authors:** Mohanad El-Harbawi, Brahim Belhaouari Samir, Lahssen El blidi, Ouahid Ben Ghanem

In the LFL prediction accuracy and validation subsection of the Results and discussion section, there is an error in Eq. (12). The correct Eq. (12) is: LFL = 0.5644 + 12.5173 (SIC0) ˗ 2.2022 (AAC + PW5) + 0.5902 (AAC) (AAC + PW5) ˗ 4.766 (SIC0) (AAC + PW5) ˗ 36.902 (PW5^2^) (AAC + PW5) + 4.4465 (SIC0^2^) (AAC + PW5) + 2.1331 (SIC0^2^) (SIC0 + GATS1v) ˗ 4.3963 (SIC0^3^) (SIC0 + GATS1v) + 215.3612 (PW5^3^) (AAC + PW5) + 0.0181 (AAC +PW5) (SIC0^3^) ˗ 20.4864 (SIC0+PW5) (PW5^2^) + 11.0002 (SIC0+GATS1v) (PW5^2^).

Eq. (12) also appears incorrectly in S3 Table. It should read: LFL = 0.5644 + 12.5173 (SIC0) ˗ 2.2022 (AAC + PW5) + 0.5902 (AAC) (AAC + PW5) ˗ 4.766 (SIC0) (AAC + PW5) ˗ 36.902 (PW5^2^) (AAC + PW5) + 4.4465 (SIC0^2^) (AAC + PW5) + 2.1331 (SIC0^2^) (SIC0 + GATS1v) ˗ 4.3963 (SIC0^3^) (SIC0 + GATS1v) + 215.3612 (PW5^3^) (AAC + PW5) + 0.0181 (AAC +PW5) (SIC0^3^) ˗ 20.4864 (SIC0+PW5) (PW5^2^) + 11.0002 (SIC0+GATS1v) (PW5^2^). The table of coefficients in S3 Table contains errors as well. Please see the correct S3 Table here.

## Supporting information

S3 Table Results comparison for the Pan et al. [37] dataset. (XLS)
